# HbA1c, lipid profiles and risk of incident type 2 Diabetes in United States Veterans

**DOI:** 10.1371/journal.pone.0203484

**Published:** 2018-09-13

**Authors:** P. Jordan Davis, Mengling Liu, Scott Sherman, Sundar Natarajan, Farrokh Alemi, Ashley Jensen, Sanja Avramovic, Mark D. Schwartz, Richard B. Hayes

**Affiliations:** 1 Department of Population Health, NYU School of Medicine, New York, NY, United States of America; 2 VA New York Harbor Healthcare System, New York, NY, United States of America; 3 George Mason University, Fairfax, VA, United States of America; University of Insubria, ITALY

## Abstract

United States Veterans are at excess risk for type 2 diabetes, but population differentials in risk have not been characterized. We determined risk of type 2 diabetes in relation to prediabetes and dyslipidemic profiles in Veterans at the VA New York Harbor (VA NYHHS) during 2004–2014. Prediabetes was based on American Diabetes Association hemoglobin A1c (HbA1c) testing cut-points, one of several possible criteria used to define prediabetes. We evaluated transition to type 2 diabetes in 4,297 normoglycemic Veterans and 7,060 Veterans with prediabetes. Cox proportional hazards regression was used to relate HbA1c levels, lipid profiles, demographic, anthropometric and comorbid cardiovascular factors to incident diabetes (Hazard Ratio [HR] and 95% confidence intervals). Compared to normoglycemic Veterans (HbA1c: 5.0–5.6%; 31–38 mmol/mol), risks for diabetes were >2-fold in the moderate prediabetes risk group (HbA1c: 5.7–5.9%; 39–41 mmol/mol) (HR 2.37 [1.98–2.85]) and >5-fold in the high risk prediabetes group (HbA1c: 6.0–6.4%; 42–46 mmol/mol) (HR 5.59 [4.75–6.58]). Risks for diabetes were increased with elevated VLDL (≥40mg/dl; HR 1.31 [1.09–1.58]) and TG/HDL (≥1.5mg/dl; HR 1.34 [1.12–1.59]), and decreased with elevated HDL (≥35mg/dl; HR 0.80 [0.67–0.96]). Transition to diabetes in Veterans was related in age-stratified risk score analyses to HbA1c, VLDL, HDL and TG/HDL, BMI, hypertension and race, with 5-year risk differentials of 62% for the lowest (5-year risk, 13.5%) vs. the highest quartile (5-year risk, 21.9%) of the risk score. This investigation identified substantial differentials in risk of diabetes in Veterans, based on a readily-derived risk score suitable for risk stratification for type 2 diabetes prevention.

## Introduction

Type 2 diabetes is a chronic metabolic disorder defined by persistent hyperglycemia due to increased insulin resistance and/or impaired insulin secretion [1–2]. In the United States, more than 30 million people (9.4% of the population) had type 2 diabetes in 2015 [3]. With aging and increasing rates of obesity, prevalence of type 2 diabetes is also rising and is currently the seventh leading cause of death [3]. Furthermore, type 2 diabetes leads to greater risk for cardiovascular disease and other conditions, such as blindness, kidney failure, and lower limb amputations, and is associated with greatly reduced overall life expectancy [1–4]. Nearly a quarter of U.S. Veterans have type 2 diabetes, more than double the prevalence of the general population [3,5–6].

Prediabetes describes individuals at increased risk for future development of type 2 diabetes [5,7]. Those with prediabetes have higher than normal blood glucose concentrations, but lower than diabetic diagnostic criteria [1,5,7–8]. Since the early 2000s, the American Diabetes Association has recommended assessment of glycated hemoglobin (HbA1c), a type of hemoglobin that indicates an individual’s three-month average plasma glucose concentration [9], as an alternative to glucose tolerance testing for diagnostic surveillance of diabetes and prediabetes. Despite the higher prevalence of type 2 diabetes among Veterans, little is known about the prevalence of prediabetes among Veterans and its impact on their development of type 2 diabetes [10].

Epidemiologic studies have shown that dyslipidemic profiles are independent risk factors for type 2 diabetes and can manifest prior to development of the disease [11–12]. Furthermore, dyslipidemia can cause decreased pancreatic beta cell function and survival particularly among diabetic patients [11,13–14]. Additional research is needed to examine the effects of lipid patterns in prediabetic and nondiabetic individuals on risk for incident type 2 diabetes.

The relationship of HbA1c, dyslipidemias and other risk factors to type 2 diabetes occurrence in Veterans is incompletely understood. Therefore, we investigated the association of HbA1c levels and associated lipid profiles with development of incident type 2 diabetes among Veterans at the Veterans Affairs New York Harbor Healthcare System (VA NYHHS).

## Materials and methods

### Data sources

Data for this study were obtained via access to the national Corporate Data Warehouse (CDW) of the Department of Veterans Affairs (VA) Office of Information and Technology (Washington D.C.). VA patient medical and administrative data are accessible from the VINCI Workspace (Veterans Affairs Informatics and Computing Infrastructure), a VA-hosted computing environment, through a secure gateway (firewall) using a Remote Desktop Connection (RDC) [15–16]. VINCI provides a centralized, secure development and research platform for conducting VA studies and allows for data storage, extraction, processing and analysis [16]. Access to linked patient level data was obtained through the Veterans Health Administration (VHA), National Data Services (NDS) and the VA Information Resource Center (VIReC), one of three Health Services Research and Development Service (HSR&D) resource centers that authorize and maintain research access to patient data and resources for research conducted within the VA [15–16].

Data extracted for use in this study in the VINCI workspace included information on patient demographics, primary care visits, laboratory data, pharmacy claims data, medical diagnostic and procedures data as ICD-9 codes, vital status and mortality records. Demographic information was based on self-report by patients and/or VA employee recorded at baseline and grouped based on existing standardized categories used in VA medical records [10,15–17]. Covariates included race (white, or other), ethnicity (not Hispanic or Latino/ other [declined/unknown], Hispanic or Latino), marital status (never married, married, separated, divorced, widowed), smoking status (never or ever smoker), body mass index ([BMI] calculated as the average weight (kg) during the baseline year or year prior to entry, divided by height (m^2^) and categorized [<18.5, 18.5–24.9, 25–29.9, ≥30]). Medical record information was also extracted for history of cardiovascular disease at baseline, defined as having ischemic heart disease (IHD; ICD-9: 410–414), cerebrovascular accident (CVA; ICD-9: 430–434), congestive heart failure (CHF; ICD-9: 428), or peripheral vascular disease (PVD; ICD-9: 443), and for history of hypertension (baseline BP ≥140/90; HTN; ICD-9:401). Systolic blood pressure (mmHg) and diastolic blood pressure (mmHg) were included as the average measurement taken during the baseline year or year prior. Data for lipid profiles were average annual fasting measurements taken during the baseline year or the year prior. This included low-density lipoprotein-cholesterol (LDL-C) (≥130, <130 mg/dl), high-density lipoprotein-cholesterol (HDL-C) (≥35, <35 mg/dl), total cholesterol (TC) (≥200, <200 mg/dl), triglyceride (TG) (≥150, <150 mg/dl), very low-density lipoprotein–cholesterol (VLDL) (≥40, <40 mg/dl) calculated as triglycerides/5 according to the Friedewald equation [18], TG/HDL ratio (≤1.5, >1.5 mg/dl), TC/HDL ratio (≥5.0, <5.0 mg/dl), and LDL/HDL ratio (≥4.0, <4.0 mg/dl).

Prediabetes was defined as hemoglobin A1c (HbA1c) of 5.7–6.4% (39–46 mmol/mol), based upon American Diabetes Association criteria, one of several possible definitions for prediabetes [1]. We further defined HbA1c of 5.0–5.6% (31–38 mmol/mol) as normoglycemic and divided prediabetes as prediabetic “moderate” risk (HbA1c: 5.7–5.9% or 39–41 mmol/mol) and prediabetic “high” risk groups (HbA1c: 6.0–6.4% or 42–46 mmol/mol), based on other research to assess predictiveness for transition from prediabetes to diabetes [1–2,5,7,19]. A diagnosis of type 2 diabetes was defined as having ≥2 ICD-9 diagnosis codes (250, 357.2, 362.0, 366.4) from clinical encounters and/or ≥2 prescriptions of DM medications other than metformin, based on the VA Primary Care Almanac approach [17,20]. This method of type 2 diabetes status ascertainment has been previously validated at the VA [10].

### Study population

Among Veterans enrolled in primary care from January 1, 2004 to December 31, 2014, at the Veterans Affairs New York Harbor Healthcare System (VA NYHHS), we identified 14,361 Veterans from the CDW, ≥18 years of age, who received at least 2 HbA1c tests and had no prior history of diabetes. After exclusion of 1,549 subjects (10.8%) with missing data, there remained 12,812 subjects for study.

### Statistical analysis

Individuals were classified at baseline according to the average of their first two HbA1c results: low glycemia <5.0% (<31 mmol/mol), normoglycemia 5.0–5.6% (31–38 mmol/mol), prediabetes moderate risk 5.7–5.9% (39–41 mmol/mol), prediabetes high risk 6.0–6.4% (42–46 mmol/mol) or type 2 diabetes risk range ≥6.5% (≥48 mmol/mol). Frequencies and proportions within these groups were calculated and the χ^2^ test and one-way ANOVA test was used to compare proportions for categorical and continuous variables across all groups. Crude type 2 diabetes incidence rates (IR) were calculated based on the number of type 2 diabetes cases and respective person-time at risk for study participants. Kaplan-Meier method and survival curves were used to calculate survival probability over time, with respect to type 2 diabetes incidence stratified by HbA1c category [21].

Cox proportional hazards models were used to assess the risk of developing incident type 2 diabetes, after adjustment for covariates. For assessment of type 2 diabetes risk, subjects were categorized by HbA1c level as <5.0% (<31 mmol/mol), 5.7–5.9% (39–41 mmol/mol), and 6.0–6.4% (42–46 mmol/mol), and were compared to those categorized as 5.0–5.6% (31–38 mmol/mol), as the reference category (Subjects in the diabetic risk range for HbA1c ≥6.5% (≥48 mmol/mol) at study entry were excluded from the analyses of type 2 diabetes incidence). Time to incident type 2 diabetes was based on the interval in days between the second HbA1c test (date of entry), subsequent to January 1, 2004, and date of the occurrence of type 2 diabetes. Subject follow-up was censored at the date of death or the end of the follow-up period, December 31, 2014, whichever came first. Estimates of HRs for all models were based on Sandwich variance estimates and ties were handled using the Efron method [21]. All models controlled for sex, race, ethnicity, marital status, BMI, smoking status, HTN, CHF, CVA, IHD, PVD, LDL, HDL, TC, TG, VLDL, TG/HDL, TC/HDL, and LDL/HDL. The proportional hazards assumption was verified by graphs of the Kaplan-Meier estimates of the survival function, log-(-)log plots, including a product term of the variables with log(time) in the Cox model and by using the proportionality test statement to assess all variables. All models were stratified by age (18–54, 55–64, ≥65) [21].

To develop estimates of the 5-year risk for incident type 2 diabetes, we constructed a 5-year risk equation [22–23]. We carried out backward elimination in the dataset with all predictors included in the stratified Cox regression analysis (p<0.10 for stay) to identify the subset of variables most strongly predictive of risk [21]. Use of forward elimination and stepwise selection methods yielded similar results. Variables selected in the final model included, HbA1c (%), body mass index (BMI), hypertension (HTN), race, VLDL, HDL and TG/HDL ratio denoted by X_1_-X_7_. HbA1c and BMI were treated as continuous variables based on the average of two HbA1c test results and BMI values at baseline. The overall C index is used as an extension of the area under the receiver operating characteristic curve (aROC) and therefore was calculated for overall ability of the risk equation to discriminate between those who develop type 2 diabetes from those who did not [24].

The risk equation developed from these findings includes X_1_-X_7_ as the baseline predictors with estimated coefficients expressed as β_1_-β_7_ for modeling the 5-year risk equation [22–23]. To account for potential violations of the proportional hazards assumptions due to age, age-stratified Cox proportional hazards models were used to derive the estimates of hazard ratios and baseline hazard functions. The mean risk score and baseline survival S_0_(5) was assessed at 5-years for each age stratified category when all factors were equal to their mean.

All data analyses were conducted using SAS 9.1 (SAS Institute, Cary, NC), SAS Enterprise Guide 6.1 (Cary, NC), and SQL Server Management Studio (Microsoft, 2014). Significance level was set at α = 0.05.

## Results

### HbA1c and type 2 diabetes risk

The study population of 12,812 adult Veterans not known to have type 2 diabetes was predominantly white (54.5%), male (95.8%), not Hispanic or Latino (84.9%), and greater than 55 years of age (66.6%). Many of these individuals were overweight (BMI: 25–29.9) (39.6%) or obese (BMI: ≥30) (38.8%) and had hypertension (77.4%) where those with a HbA1c ≥5.7% had higher percentages of these conditions (**Table 1**). Based on HbA1c, 33.5% of these subjects were normoglycemic, 28% were in the moderate prediabetic risk range, 27.1% were in the high prediabetic risk range, 5.9% had low glycemia and 5.4% were in the diabetic risk range.

**Table 1:** Demographic and clinical covariate data according to HbA1c status.

	Low Glycemia		Normoglycemia		Moderate PreDM		High PreDM		DM Risk		P-value
	< 5.0%		5.0–5.6%		5.7–5.9%		6.0–6.4%		≥ 6.5%		
n (%)	760	5.9	4,297	33.5	3,587	28.0	3,473	27.1	695	5.4	
Age (years)											<0.0001
18–54	345	45.4	1561	36.3	1082	30.2	1054	30.4	246	35.4	
55–64	226	29.7	1363	31.7	1176	32.8	1162	33.5	217	31.2	
≥65	189	24.9	1373	32	1329	37.1	1257	36.2	232	33.4	
Sex											<0.0001
F	28	3.7	236	5.5	129	3.6	114	3.3	27	3.9	
M	732	96.3	4061	94.5	3458	96.4	3359	96.7	668	96.1	
Race											<0.0001
White	331	43.6	2614	60.8	2053	57.2	1669	48.1	310	44.6	
Other	429	56.5	1683	39.2	1534	42.8	1804	51.9	385	55.4	
Ethnicity											0.2004
Not Hisp/Lat	635	83.6	3617	84.2	3078	85.8	2960	85.2	583	83.9	
Hisp/Lat	125	16.5	680	15.8	509	14.2	513	14.8	112	16.1	
Marital Status											<0.0001
Never Married	206	27.1	1124	26.2	789	22	734	21.1	134	19.3	
Married	222	29.2	1477	34.4	1405	39.2	1398	40.3	273	39.3	
Separated	62	8.2	264	6.1	226	6.3	226	6.5	69	9.9	
Divorced	206	27.1	994	23.1	771	21.5	734	21.1	148	21.3	
Widowed	64	8.4	438	10.2	396	11	381	11	71	10.2	
Smoking Status											0.0012
Never	159	20.9	792	18.4	666	18.6	673	19.4	91	13.1	
Ever	601	79.1	3505	81.6	2921	81.4	2800	80.6	604	86.9	
BMI (kg/m^2^)											<0.0001
<18.5	5	0.7	35	0.8	25	0.7	24	0.7	3	0.4	
18.5–24.9	209	27.5	1090	25.4	732	20.4	571	16.4	77	11.1	
25–29.9	304	40	1770	41.2	1447	40.3	1315	37.9	235	33.8	
≥30	242	31.8	1402	32.6	1383	38.6	1563	45	380	54.7	
HTN											<0.0001
No	233	30.7	1147	26.7	754	21	606	17.5	143	20.6	
Yes	527	69.3	3150	73.3	2833	79	2867	82.6	552	79.4	
CHF											0.1388
No	756	99.5	4247	98.8	3541	98.7	3417	98.4	685	98.6	
Yes	4	0.5	50	1.2	46	1.3	56	1.6	10	1.4	
CVA											0.0098
No	735	96.7	4145	96.5	3428	95.6	3303	95.1	674	97	
Yes	25	3.3	152	3.5	159	4.4	170	4.9	21	3.0	
IHD											<0.0001
No	610	80.3	3235	75.3	2512	70	2400	69.1	490	70.5	
Yes	150	19.7	1062	24.7	1075	30	1073	30.9	205	29.5	
PVD											0.0034
No	707	93	3922	91.3	3256	90.8	3102	89.3	639	91.9	
Yes	53	7	375	8.7	331	9.2	371	10.7	56	8.1	
Blood Pressure											
SBP (mmHg)	125.8	±14.7	125.9	±14.3	126.8	±13.7	127.4	±13.6	129.9	±15.0	0.0035
DBP (mmHg)	75	±9.2	74.8	±9.7	74.7	±9.2	75.2	±9.4	76.3	±9.9	0.0089

Data are shown as n(%) or (±SD). Prediabetes (PreDM), Diabetes (DM), Body Mass Index (BMI), Hypertension (HTN), Congestive Heart Failure (CHF), Cerebrovascular Accident (CVA), Ischemic Heart Disease (IHD), Peripheral Vascular Disease (PVD), Systolic Blood Pressure (SBP), Diastolic Blood Pressure (DBP)

Excluding 695 subjects with HbA1c in the diabetic range at study entry, we identified 1,270 incident cases of type 2 diabetes in 12,117 nondiabetic study participants followed on average for 3.6 years (**Table 2**). Type 2 diabetes incidence rates increased across the range of HbA1c, from 1.08 (per 100 person-years [0.76–1.53]) for those with low glycemia to 6.41 (per 100 person-years [5.96–6.89]) among those in the high prediabetic range. Both the prediabetic moderate risk (HR 2.37 [1.98–2.85]) and the prediabetic high risk groups (HR 5.59 [4.75–6.58]) showed significantly increased type 2 diabetes risk compared to the normoglycemic reference group, after adjustment for other factors (**Table 2**, Model 2). Type 2 diabetes risks were nearly the same for those in the low glycemia and normoglycemic categories of HbA1c.

**Table 2:** Crude incidence rates and Cox proportional hazards models: risk of type 2 diabetes according to levels of HbA1c.

		Incidence Rate^†^				Model 1*			Model 2^§^		
Glycemia Group	HbA1c (%)	Cases	PY	IR	95%CI	HR	95%CI	P-value	HR	95%CI	P-value
Low Glycemia	<5.0	32	2966	1.08	(0.76–1.53)	0.94	(0.65–1.36)	0.7276	0.95	(0.65–1.37)	0.7786
Normoglycemia	5.0–5.6	179	16207	1.10	(0.95–1.28)	Ref			Ref		
PreDM: Moderate	5.7–5.9	322	12488	2.58	(2.31–2.88)	2.55	(2.13–3.06)	<0.0001	2.37	(1.98–2.85)	<0.0001
PreDM: High	6.0–6.4	737	11500	6.41	(5.96–6.89)	6.15	(5.23–7.23)	<0.0001	5.59	(4.75–6.58)	<0.0001

^†^Unadjusted Incidence Rate (IR, per 100 PY), Person-Year (PY). Those with ≥2 HbA1c tests in the DM range (≥6.5%) were excluded from analyses of incident diabetes, as by definition they would be classified as having diabetes. Prediabetes (PreDM).

*Model 1: Stratified by age; Adj for: sex, race, ethnicity, marital status.

^§^Model 2: Stratified by age; Adj for: sex, race, ethnicity, marital status, BMI, smoking status, HTN, CHF, CVA, IHD, PVD, LDL, HDL, TC, TG, VLDL, TG/HDL, TC/HDL, LDL/HDL.

### Dyslipidemias and type 2 diabetes risk

Incident type 2 diabetes was also assessed by lipid categories. A majority of study subjects had levels of LDL <130 mg/dl (82.4%), HDL ≥35 mg/dl (90.7%), TC<200 mg/dl (74.8%), TG<150 mg/dl (73.1%), VLDL<40 mg/dl (87.8%), TG/HDL >1.5 mg/dl (74.7%), TC/HDL<5.0 mg/dl (88.6%) and LDL/HDL <4.0 mg/dl (97.7%). Cox proportional hazards models showed significantly increased risks for type 2 diabetes with respect to VLDL ≥40 mg/dl (HR 1.31 [1.09–1.58]), TG/HDL ≥1.5 mg/dl (HR 1.34 [1.12–1.59]), while a decreased risk was observed for HDL ≥35 mg/dl (HR 0.80 [0.67–0.96]) after adjustment for all other factors. Increased risks were observed for other lipid factors, but were not statistically significant (**Table 3**).

**Table 3:** Cox proportional hazards models: risk of type 2 diabetes according to lipid profiles.

	Lipid Profiles		Model*		
	N	%	HR	95% CI	P-value
LDL (mg/dl)					
<130	9987	82.4	Ref		
≥130	2130	17.6	1.03	(0.84–1.26)	0.7639
HDL (mg/dl)					
<35	1131	9.3	Ref		
≥35	10986	90.7	0.8	(0.67–0.96)	0.0155
TC (mg/dl)					
<200	9059	74.8	Ref		
≥200	3058	25.2	0.92	(0.77–1.11)	0.4046
TG (mg/dl)					
<150	8853	73.1	Ref		
≥150	3264	26.9	1.08	(0.92–1.27)	0.3476
VLDL (mg/dl)					
<40	10637	87.8	Ref		
≥40	1480	12.2	1.31	(1.09–1.58)	0.0038
TG/HDL (mg/dl)					
≤1.5	3069	25.3	Ref		
>1.5	9048	74.7	1.34	(1.12–1.59)	0.0012
TC/HDL (mg/dl)					
<5.0	10729	88.6	Ref		
≥5.0	1388	11.5	0.87	(0.72–1.06)	0.1692
LDL/HDL (mg/dl)					
<4.0	11841	97.7	Ref		
≥4.0	276	2.3	1.32	(0.97–1.79)	0.0731

*Model: Hazard Ratio (HR). Stratified by age; Adj for: sex, race, ethnicity, marital status, BMI, smoking status, HTN, CHF, CVA, IHD, PVD, LDL, HDL, TC, TG, VLDL, TG/HDL, TC/HDL, LDL/HDL. Those with ≥2 HbA1c tests in the DM range (≥6.5%) were excluded from analyses of incident diabetes as by definition they would be classified as having diabetes. Low-density lipoprotein (LDL), very low-density lipoprotein (VLDL), high-density lipoprotein (HDL), triglyceride (TG), total cholesterol (TC).

### Sensitivity analyses

To investigate possible effects on study results from the implementation of new clinical practice guidelines for HbA1c use at the VA, we compared analyses of data from 2011–2014 (after implementation of the new HbA1c practice guidelines) to results from 2004–2008 (pre-implementation). We also assessed impact of more restrictive eligibility criteria of having had 5 or more HbA1c tests vs. our criteria of 2 or more tests. Associations observed overall, were similar to those found in subset analyses (**S1 Appendix**).

### Type 2 diabetes risk prediction

Based on the backward elimination method from a fully adjusted model (**Table 2**, Model 2), we developed an age-stratified 5-year risk prediction model for transition to type 2 diabetes (**Table 4**). For example, a 0.1-unit increase in HbA1c is associated with a 1.33 increase in the HR and a one unit increase in BMI is associated with a more modest 1.03 increase in the HR, each after adjusting for other factors. The C statistic calculated for overall discrimination for the model was good at 0.70 (95% CI: 0.65–0.74) [24]. Based on an age-stratified risk prediction model including HbA1c, VLDL, HDL and TG/HDL, BMI, hypertension and race (**S2 Appendix**), there is an 18.6% average potential 5-yr risk for incident type 2 diabetes among prediabetic Veterans. For individuals in the population, there is a 5-year risk differentials of 62% for the lowest (5-year risk, 13.5%) vs. the highest quartile (5-year risk, 21.9%) of the risk score. Global models including all interaction terms demonstrated similar model fit (AIC = 22749.2, SBC = 22983.579) compared to models without interaction terms (AIC = 22746.05, SBC = 22909.57), suggesting that the more conservative model without interaction terms is suitable.

**Table 4:** Cox proportional hazards 5-year prediction model for incident type 2 diabetes.

Backward Elimination Final Model Selection^*^						
Parameter	HR	95% CI	Estimate	SE	P-value	Values used in Risk Equation
HbA1c	1.33	(1.29–1.38)	0.28822	0.01731	<0.0001	Per 0.1 unit
BMI	1.03	(1.02–1.04)	0.02535	0.00491	<0.0001	Per 1 unit
HTN	1.20	(1.01–1.43)	0.18203	0.09015	0.0435	1 for Yes, 0 for No
Race	1.24	(1.09–1.42)	0.21498	0.06746	0.0014	1 for White, 0 otherwise
VLDL	1.39	(1.18–1.63)	0.32880	0.08080	<0.0001	1 for ≥40, 0 for <40
HDL	1.48	(1.25–1.75)	0.39066	0.08609	<0.0001	1 for <35, 0 for ≥35
TG/HDL	1.47	(1.22–1.78)	0.38673	0.09540	<0.0001	1 for >1.5, 0 for ≤1.5
**5-year Baseline Survival**^§^**: Stratified by Age**						
Age (years)		S_0_(5)		95% CI		Mean Risk Score ($\beta_{i}{\bar{x}}_{i}$)
18–54		0.82077		(0.80–0.85)		3.09574
55–64		0.81419		(0.80–0.83)		3.16604
≥65		0.84106		(0.82–0.86)		3.09643

*Hazard Ratio (HR), Hemoglobin A1c (HbA1c), Body Mass Index (BMI), Hypertension (HTN), very low-density lipoprotein (VLDL), high-density lipoprotein (HDL), triglyceride (TG). The final model has been stratified by age categories. ^§^Baseline survival S_0_(5) at 5-years for each age stratified category when all factors were equal to the mean.

## Discussion

Our research in a large cohort of Veterans in New York City shows that prediabetic Veterans classified as having a moderate risk for type 2 diabetes have a more than 2-fold increase in risk of incident type 2 diabetes, and that Veterans classified at high risk have a more than 5-fold increase in risk for this disease. Our study also identified associations of selected cardiovascular risk factors with modest changes in type 2 diabetes risk. Furthermore, our research provides a useful prediabetes specific risk equation for the estimation of 5-year risk of incident type 2 diabetes in this cohort, demonstrating stratification of individuals over a broad risk range, based on selected demographic and clinical factors. The HbA1c test used in conjunction with lipid profiles and other factors in the proposed risk equation, serves as a simple tool to predict risk of incident type 2 diabetes in this population.

Our research is the first systematic evaluation in Veterans of type 2 diabetes risk associated with HbA1c, as the introduction of screening with HbA1c for prediabetes and type 2 diabetes at the VA is relatively recent [17]. Our study showed the importance of examining moderate versus high prediabetes risk groups and highlights the high prevalence of prediabetes at the VA where more than half of all Veterans tested have this condition. Efforts to mitigate transition of this high risk state to incident type 2 diabetes are of critical importance within the older and sicker Veteran population.

Dyslipidemia is a major risk factor for cardiovascular disease, particularly among diabetics, which remains the number one cause of death in the United States [25–26]. The characteristic features of dyslipidemia, such as increased triglycerides and LDL levels combined with low HDL, are metabolically interrelated and begin occurring several years before the onset of type 2 diabetes and, as we and others show, may serve as independent risk factors for the condition [11–12,27]. Additionally, increased production of VLDL plasma levels by hepatocytes significantly contribute and are thought to initiate a cascade of progressively abnormal levels of atherogenic lipids [27].

Hypercholesterolemia also contributes to decreased insulin production and pancreatic beta cell dysfunction [28–29]. Increasing cholesterol and LDL levels related to beta cell functional decline were found to be higher in prediabetic and diabetic individuals [30–31]. Cholesterol homeostasis is therefore an important factor in delaying or preventing insulin secretory defects [13–14,28,31]. A recent study using mouse models has also demonstrated the pathogenesis of beta cell dysfunction and insulin resistance by presence of lipoproteins [11]. Increased triglyceride levels were shown to alter insulin secretion along inflammatory free fatty acid metabolic pathways and influence pancreatic beta cell function and survival [14,31–32].

High-density lipoproteins, on the other hand, stimulate insulin secretion, inhibit beta cell apoptosis [11,33] and may also enhance skeletal muscle glucose uptake [34]. Conversely, increased BMI and atherogenic lipids in prediabetes and type 2 diabetes adds metabolic stress that interferes with HDL production, thereby exacerbating pancreatic cell dysfunction and insulin resistance [11]. Decreased HDL levels may therefore worsen glycemic control and accelerate progression to type 2 diabetes in prediabetic individuals [35], although not all studies agree [36].

Our study found increased type 2 diabetes risk with increasing VLDL and TG/HDL, while a decreased risk was seen for elevated HDL. While TG/HDL ratios predict for cardiovascular disease, additional data is needed regarding prediction of type 2 diabetes risk among prediabetics and normoglycemic individuals [37–39]. TG/HDL has been related to insulin resistance [38] and type 2 diabetes [40] consistent with our findings.

Strengths of this study include a large population at the VA NYHHS, use of robust electronic medical record information and survival analysis to model relevant outcomes and a validated methodology for case ascertainment [10,15–17,20]. The move toward use of HbA1c in recent years, has gained traction due to several advantages as a diagnostic test [1–3,7]. HbA1c testing is now better standardized as a diagnostic test in the U.S. and has less intraindividual biologic variability compared to fasting plasma glucose (FPG) [1–2,7]. It also provides a better measure of overall levels of glycemia by reflecting longer-term exposure intervals (~3 months, the average half-life of a red blood cell), has the practical advantage of not requiring fasting at collection, higher repeatability and serves as a better guide to clinical management [1,3,7,9–10,17].

A limitation in HbA1c testing to distinguish normoglycemic from prediabetic subjects (or moderate from high risk prediabetics) is that the cutpoints for these groups are somewhat arbitrary, with different research groups using slight modifications of the definitions we employed [1–2,5,7,19]. In fact, prediabetes and associated HbA1c (or other measures of glycemic state, such as fasting glucose) are on a continuum from health to disease state and may also have different implications for people with varying profiles of other diabetes risk factors. Also, we used cut-points for HDL (≥35 versus <35 mg/dl), as recommended by the National Institute of Diabetes and Digestive and Kidney Diseases (NIDDK) and ADA for diabetes risk evaluation in overweight adults [41–43], recognizing that optimum targets of HDL levels >40 mg/dl for men and >50 mg/dl for women have also been suggested [44–45]. Although the Veterans Administration provides a rich resource for research, there are also certain limitations to use of data from this source. We did not have information on diet or physical activity of study participants, nor did we evaluate the impact of medication use, which is a topic of ongoing research. We did not consider the potential influence of medication use on diabetes risk, although this could be dealt with in detail in subsequent research on this population. Other limitations of our study include the predominately white male and older study population, and possible selection bias of patients given HbA1c testing at the VA, although our sensitivity analysis by time period and frequency of pre-entry HbA1c tests showed similar associations when more restrictive eligibility criteria were used. Additionally, intraindividual variation in fasting lipid levels may contribute to potential misclassification of individuals on dyslipidemia profiles.

## Conclusions

This study demonstrates the importance of HbA1c screening at the VA to identify those at greater risk for incident type 2 diabetes. Results also add evidence on the importance of lipid components as independent risk factors for type 2 diabetes. Findings suggest that prevention strategies should target prediabetic Veterans with HbA1c levels ≥5.7% (42 mmol/mol) and prediabetic dyslipidemia to reduce incident type 2 diabetes among Veterans.

## Supporting information

S1 Appendix Sensitivity analyses. (DOCX)

S2 Appendix Predictive model for 5-year percent risk of diabetes. (DOCX)
