# Supplementary material for: HbA1c, lipid profiles and risk of incident type 2 Diabetes in United States Veterans
Source: PLoS One. 2018 Sep 13;13(9):e0203484. doi: 10.1371/journal.pone.0203484 (PMC6136717; doi:10.1371/journal.pone.0203484)
Supplement: S1 Appendix — (DOCX) [file pone.0203484.s001.docx]

**S1 Appendix**: Sensitivity Analyses

|  | | | | | | | | | | | | | | |
| --- | --- | --- | --- | --- | --- | --- | --- | --- | --- | --- | --- | --- | --- | --- |
| **Cox Proportional Hazards Models: Risk of Type 2 Diabetes According to Levels of HbA1c in Various Population Subsets** | | | | | | | | | | | | | | |
| Low Glycemia | | |  | Normoglycemia | | |  | PreDM: Moderate | | |  | PreDM: High | | |
| <5.0% | | |  | 5.0-5.6% | | |  | 5.7-5.9% | | |  | 6.0-6.4% | | |
|  | | | | | | | | | | | | | | |
| **Cohort: 2004-2014: ≥1 annual PC visits and ≥1 HbA1c tests^*^ (N=30,925).** | | | | | | | | | | | | | | |
| HR | 95% CI | P-value |  | HR | 95% CI | P-value |  | HR | 95% CI | P-value |  | HR | 95% CI | P-value |
| 1.07 | (0.82-1.39) | 0.6351 |  | Ref | | |  | 2.41 | (2.11-2.75) | <0.0001 |  | 6.47 | (5.74-7.29) | <0.0001 |
|  |  |  |  |  |  |  |  |  |  |  |  |  |  |  |
| **Cohort: 2004-2014: ≥5 annual PC visits and ≥2 HbA1c tests^*^ (N=10,928).** | | | | | | | | | | | | | | |
| HR | 95% CI | P-value |  | HR | 95% CI | P-value |  | HR | 95% CI | P-value |  | HR | 95% CI | P-value |
| 0.89 | (0.61-1.29) | 0.5314 |  | Ref | | |  | 2.23 | (1.86-2.66) | <0.0001 |  | 5.26 | (4.47-6.19) | <0.0001 |
|  |  |  |  |  |  |  |  |  |  |  |  |  |  |  |
| **Cohort: 2004-2008: ≥2 annual PC visits and ≥2 HbA1c tests^*^ (N=7,834).** | | | | | | | | | | | | | | |
| HR | 95% CI | P-value |  | HR | 95% CI | P-value |  | HR | 95% CI | P-value |  | HR | 95% CI | P-value |
| 1.06 | (0.72-1.55) | 0.7782 |  | Ref | | |  | 2.52 | (2.06-3.08) | <0.0001 |  | 5.81 | (4.86-6.96) | <0.0001 |
|  |  |  |  |  |  |  |  |  |  |  |  |  |  |  |
| **Cohort: 2011-2014: ≥2 annual PC visits and ≥2 HbA1c tests^*^ (N=7,900).** | | | | | | | | | | | | | | |
| HR | 95% CI | P-value |  | HR | 95% CI | P-value |  | HR | 95% CI | P-value |  | HR | 95% CI | P-value |
| 0.73 | (0.22-2.39) | 0.5974 |  | Ref | | |  | 1.87 | (1.17-2.98) | 0.0087 |  | 6.64 | (4.35-10.12) | <0.0001 |
|  |  |  |  |  |  |  |  |  |  |  |  |  |  |  |
| ^*^Models: Hazard Ratio (HR), Stratified by age; Adj for: sex, race, ethnicity, marital status, BMI, smoking status, HTN, CHF, CVA, IHD, PVD, LDL, HDL, TC, TG, VLDL, TG/HDL, TC/HDL, LDL/HDL. Those with ≥2 A1c tests in the DM range (≥6.5%) were excluded from analyses of incident diabetes as by definition they would be classified as having diabetes. Prediabetes (PreDM). | | | | | | | | | | | | | | |
