# Supplementary material for: HbA1c, lipid profiles and risk of incident type 2 Diabetes in United States Veterans
Source: PLoS One. 2018 Sep 13;13(9):e0203484. doi: 10.1371/journal.pone.0203484 (PMC6136717; doi:10.1371/journal.pone.0203484)
Supplement: S2 Appendix — (DOCX) [file pone.0203484.s002.docx]

**S2 Appendix:** Predictive model for 5-year percent risk of diabetes.

Where β_i_ is the estimated regression coefficient, X_i_ corresponds to the *i*^th^ value of each risk factor, x̄ is the corresponding mean value, and p indicates the number of risk factors (21-22). The baseline diabetes-free survival for 5 years S_0_(t) for each stratified age group S_0_(5) = 0.82077 for ages 18-54, S_0_(5) = 0.81419 for age 55-64, and S_0_(5) = 0.84106 for ages ≥65. The Mean Risk score is the value when all covariates were set to the mean for each stratified age group, where β_i_x̄_i_ = 3.09574 for ages 18-54, β_i_x̄_i_ = 3.16604 for age 55-64, and β_i_x̄_i_ = 3.09643 for ages ≥65.

5-year Diabetes Percent Risk Equation:

= 1-S_0_(t) ^exp(Risk Score - Mean Risk Score)^ x 100

= 1-S_0_(5) ^exp(ƩpβiXi – Ʃpβix̄i)^ x 100

For example, for an individual characterized as a 57-year old White male Veteran with an HbA1c of 5.9, BMI of 27, hypertension, VLDL ≥40, HDL ≥35 and TG/HDL >1.5, the 5-year Diabetes Percent Risk would have the following:

= 1-S_0_(5) ^exp [ { (β1 x HbA1c) + (β2 x BMI) + (β3 x HTN) + (β4 x Race) ) + (β5 x VLDL) + (β6 x HDL) + (β7 x TG/HDL) } – (Mean Risk Score) ]^ x 100

= 1- 0.81419 ^exp [ { (0.28822 x 5.9) + (0.02535 x 27) + (0.18203 x 1) + (0.21498 x 1) + (0.3288 x 1) + (0.39066 x 0) + (0.38673 x 1) } – (3.16604) ]^ x 100

= 25.9%

Based on calculation from the equation above, there is an 18.6% average potential 5-yr risk for incident diabetes among prediabetic Veterans, with the highest quartile (Q3) having 21.87% versus the lowest quartile (Q1) of risk at 13.54%.
